# Personal failure makes society seem fonder: An inquiry into the roots of social interdependence

**DOI:** 10.1371/journal.pone.0201361

**Published:** 2018-08-03

**Authors:** Edward Orehek, Arie W. Kruglanski

**Affiliations:** 1 Department of Psychology, University of Pittsburgh, Pittsburgh, Pennsylvania, United States of America; 2 Department of Psychology, University of Maryland, College Park, Maryland, United States of America; University of Melbourne, AUSTRALIA

## Abstract

A universal consideration among people concerns the relative premium placed on social interdependence relative to self-reliant independence. While interdependence requires submission to social constraints, it also offers empowerment through coalition. While independence fosters freedom, it also imposes individual responsibility for attained outcomes whether good or bad. In four studies we obtain the first direct evidence that failure prompts a shift toward interdependence. Implications are discussed for conditions under which people are driven to collective action.

## Introduction

A perennial puzzle in human behavior is a person’s connection to groups and a readiness to subordinate personal needs to social cooperation. An essential aspect of human nature is the tension between individuals’ independence and their interdependence with others. We crave personal autonomy but also yearn for relatedness to others that can constrain individual freedom. Individuals and societies differ in the relative value they place on these two modes of social interaction [1] and in some circumstances a wave of interdependence may rise and bring forth social movements that alter the course of history. But what accounts for a relative shift in orientation from independence to interdependence? The present work is poised to address this question.

Early in the course of psycho-social development, infants are dependent on adult caretakers and only gradually acquire a measure of autonomy. Interdependence with others continues, however, forming a delicate balance with people pining for freedom from constraints. That balance is upset by situational challenges that undermine confidence in one’s self-reliance and threaten abject failure. When this happens, a person orients toward others, willing to trade the freedoms of independence for the comforts of interdependence. Donning an interdependent orientation has an empowering effect; it augments individuals’ sense of personal significance and induces the readiness to confront all manner of adversity, including one’s own potential demise [2–6].

Prior evidence for the shift toward interdependence following failure has been suggestive, though incomplete. For instance, securely attached infants exposed to a stranger typically explore their environment when their caregiver is present, signal a desire for proximity to the caregiver when s/he departs, and actively seek proximity when s/he returns [7]. In other words, when the stranger enters, the child retreats to the parent, seeking a safe haven. This hints at their increased dependence on their social base (i.e., social interdependence) when confronted with a potentially threatening situation. As adults, individuals presented with a frightening prospect of suffering electric shock preferred to affiliate with others in a similar situation rather than coping with it alone [8]).

Beyond laboratory studies, the link between personal success or significance and independence has been observed worldwide. Wealth and individualism are positively associated across cultures [9, 1, 10]. Although the research on this association has been correlational, it has been argued that “The arrow of causality is not from individualism to wealth, but from wealth to individualism” [11]). Supporting this notion, economic development over four decades was associated with a shift toward a more individualistic orientation [12–14]. Similarly, subjective well-being was found to be positively correlated with individualism across cultures [15–16]. Thus, the sense of success and well-being (e.g. produced by wealth) seems to be associated generally with a shift toward independence and away from interdependence.

Though consistent with the notion that failure (success) causes an increase in interdependence (independence), the research to date has stopped short of directly testing this hypothesis. The developmental research [7] could be specific to infants and their unique rapport with caregivers. Affiliation in response to fear was arguably prompted by the need to resolve epistemic uncertainty through comparison with common-fate others [8]. The research on wealth, subjective well-being and individualism was correlational in nature. In summary, firm data are lacking on the implied causal link between a sense of personal failure and interdependence. The present research addresses this concern, thus filling an important gap in knowledge. To test our hypothesis, we conducted two surveys in which participants’ perceptions of personal success were measured and two experiments in which participants’ sense of personal success versus failure was induced, followed by an assessment of the relative value participants placed on interdependence versus independence.

## Present research

The present research investigates the possibility that experiences of personal failure or success will cause a shift in the prioritization of interdependence versus independence. Because the priority of a person’s goals shifts dynamically, the relative predominance of motives in a given instant determines how one would act and what one would do [17–21]. Important motives lay dormant while other important motives are pursued, and the relative priority of those motives may shift over time. The bulk of one’s attention is typically directed toward the highest priority motive and attention is directed away from lower priority motives [22–25].

These priority shifts have been shown to occur among basic motivational systems. For example, recent research has measured or manipulated the relative predominance of promotion and prevention foci [26–31], locomotion and assessment modes [32–34], and independent and interdependent mindsets [35, 6, 36–37], and observed corresponding shifts in behavior governed by the motivational systems in question. Whereas this body of research examined the *consequences* of shifts in motivational systems, the current research investigates the *antecedents* of a major such shift of present interest, namely the shift from an independent to an interdependent orientation.

### Study 1: Correlation between personal success and interdependence

Our first study was conducted as an initial test of the relationship between perceptions of personal success and interdependence. Previous research has found associations between wealth or subjective well-being and interdependence at the cultural level [15, 16, 9, 1, 10], but has not specifically assessed participants’ perceptions of success. To address this limitation, we collected such responses by asking participants to answer one question aimed at assessing their perceptions of success and one question aimed at assessing their interdependence. No other variables were measured.

#### Participants

One hundred American participants (62 males, 38 females) were sampled through Mechanical Turk in exchange for $.40. Participants were from 31 states, 18–62 years (*M* = 29.61, *SD* = 8.66). In all of our studies, data analysis occurred only after the full sample had completed participation. Participants read a consent script on the internet and clicked to continue the survey, indicating that they consented to participate. This study and informed consent procedure were approved by University of Pittsburgh’s Internal Review Board (PRO14060240).

#### Procedure and materials

**Personal success**. Respondents’ perceptions of their personal success were measured using a single item, “I have been successful in achieving my personal and individual goals.” Response options ranged from (1) strongly disagree to (7) strongly agree.

**Interdependence**. Interdependence was assessed via a single item we created. Participants were asked to choose which statement they agreed with more: “a parent’s major goal should be ensuring that their children have a good education and a chance to succeed in life,” “a parent’s major goal should be ensuring that their children serve their nation,” or “a parent’s major goal should be ensuring that their children serve their religion.” Interdependence with others manifests via attachment to social groups, and subordination of individual interests to group interests. In contrast, independence manifests via priority placed on individual interests over group interests. Therefore, we established prior to the study that those who felt that nation and religion were most important were considered to espouse interdependent views (i.e., have an interdependent identification), whereas those who felt that education and success were most important were considered to espouse independent views (i.e., have an independent identification).

#### Results and discussion

To test the prediction that perceptions of personal success would be associated with interdependent (nation or religion) rather than independent (education and success) identifications, we performed a multinomial logistic regression on identification (nation; religion; self) as predicted by personal success. The independent identification (i.e., self) served as the reference category.

Personal success accounted for significant variance in identification (*χ*^2^(2) = 14.60, *p* = .001, Cox-Snell *R*^*2*^ = .14) and we therefore proceeded with examining the separate binary logistic analyses (i.e., nation vs. self; religion vs. self). Overall classification rate was adequate with 73% of participants being classified into the correct identification based on the specified model.

Participants were 47% less likely to self-identify according to their nation compared to as an individual for each unit increase in personal success, *B* = -.63, *χ2* (1) = 9.78, *p* = .002, exp(*B*) = .53, 95% CI [.36; .79]. Similarly, participants were 45% less likely to self-identify according to their religion compared to as an individual for each unit increase in personal success, *B* = -.60, *χ2* (1) = 5.73, *p* = .02, exp(*B*) = .55, 95% CI [.34; .90].

An additional model with “religion” as the reference category indicated that personal success was not associated with differences in self-identifications according to one’s religion compared to one’s nation, *B* = -.03, *χ2* (1) = .01, *p* = .91, exp(*B*) = .97, 95% CI [.55; 1.70].

These results confirm the hypothesized link between perceptions of personal success and social interdependence. As individuals perceived their personal success less positively, they tended to also espouse interdependent views.

### Study 2: Representative samples from Egypt, Indonesia, and Pakistan

Our second study was conducted as a direct replication of Study 1 in different cultural contexts, once again investigating the relationship between perceptions of personal success and social interdependence using the same items. We used data that was collected from representative samples in Egypt, Indonesia, and Pakistan.

#### Participants

Representative samples from three countries (over 18 years of age) were obtained by using the publicly available data collected under the Public Opinion in the Islamic World on Terrorism, al Qaeda, and US Policies Survey, which can be accessed online through the START Terrorism Data Archive Dataverse. The sample included 1101 participants from Egypt, 1120 participants from Indonesia, and 1200 participants from Pakistan. The total participants sampled included 3421 citizens from these three nations (1730 male, 1691 female). The age of participants ranged from 18–80 (*M* = 36). Participants for Study 2 were read the informed consent form and verbally indicated that they consented to participate. This study and consent procedure were approved by University of Maryland’s Institutional Review Board (#01670).

#### Procedure and materials

The surveys were administered to participants during face to face interactions in the participants’ homes by the following survey companies: Emac Training and Research Center in Cairo, Egypt, A.C. Neilsen in Karachi, Pakistan, and Synovate in Jakarta, Indonesia. The surveys were translated into the local language by native speakers. Following the translation, the survey was checked by a second native speaker and any disagreements between speakers were satisfactorily resolved. Participants had the option of completing the survey in English or in the local language, but the vast majority chose to complete it in the local language.

#### Results and discussion

To test the hypothesis that perceptions of less personal success would be associated with interdependence rather than independence, we performed a multinomial logistic regression on identification (nation; religion; self) as predicted by personal success while controlling for nation (Egypt; Indonesia; Pakistan; because participants were nested within their nation, but an *N* of 3 countries would be too small to perform hierarchical linear modeling). The independent identification served as the reference category. Because the relationship between the dependent variable and the logit pertaining to the comparison between “religion” versus “self” was nonlinear, we conducted the planned analysis using bias-corrected accelerated bootstrapping [38]. All other assumptions were met. Out of 3421 respondents, 3109 answered both questions of interest for the present study, so we proceeded by analyzing their responses. The remaining 312 participants opted out of the question(s).

The two independent variables accounted for significant variance in identifications, *χ*^2^(6) = 321.68, *p* < .001, Cox-Snell *R*^*2*^ = .10. Both nationality (*χ*^2^(4) = 282.33, *p* < .001) and personal success (*χ*^2^(2) = 10.33, *p* = .01) affected individuals’ identification, so we proceeded with examining the separate binary logistic regression results (i.e., nation vs. self; religion vs. self). Overall classification rate was reasonable such that 68.3% of participants were classified into their self-selected category correctly based on the specified model.

Participants from Egypt were 86% more likely to self-identify according to their nation rather than as an individual compared to participants from Pakistan (*B* = .62, *χ2* (1) = 24.09, *p* = .001, exp(*B*) = 1.86, 95% CI [.39; .87]), whereas participants from Indonesia were 80% less likely to identify according to their nation rather than as an individual compared to participants from Pakistan (*B* = -1.60, *χ2* (1) = 80.05, *p* = .001, exp(*B*) = .20, 95% CI [-1.99; -1.28]). After adjusting for such differences in nationality, and although this effect was only marginally significant, participants were 10% more likely to identify according to their nation rather than as an individual for each unit *decrease* in personal success, *B* = .09, *χ2* (1) = 2.80, *p* = .10, exp(*B*) = 1.10, 95% CI [-.01; .19].

Participants from Egypt were 56% less likely and participants from Indonesia were 53% less likely to self-identify according to their religion rather than as an individual compared to participants from Pakistan (*B* = -.83, *χ2* (1) = 34.73, *p* = .001, exp(*B*) = .44, 95% CI [-1.10; -.58]; *B* = -.76, *χ2* (1) = 41.06, *p* = .001, exp(*B*) = .47, 95% CI [-.98; -.54]). Participants were 15% more likely to identify according to their religion rather than as an individual for each unit *decrease* in personal success, *B* = .14, *χ2* (1) = 8.99, *p* = .01, exp(*B*) = 1.15, 95% CI [.04; .23].

An additional model with “religion” as the reference category indicated that personal success did not predict differences in identifying according to one’s religion compared to one’s nation after adjusting for participants’ country of origin, *B* = -.05, *χ2* (1) = .55, *p* = .44, exp(*B*) = .95, 95% CI [-.17; .09]. This result indicates that, as expected, there was no difference between those who identified according to their nation to those to identified according to their religion.

The present study utilized different populations than the preceding study and, as such, directly speaks to the generalizability of our hypothesis. Participants who reported less personal success espoused interdependent self-views (i.e., religiosity or nationalism) to a greater extent than independent views. While Studies 1–2 provided evidence for our hypothesis using a more direct measure of participants’ sense of personal significance than what was previously available in the literature, they were correlational and hence limited in their ability to suggest a causal link between personal success and collectivism. To address this issue, our remaining studies utilized experimental designs. Another important limitation of the first two studies is that they relied on a single item measure of interdependence that has not been previously validated. Therefore, our remaining studies used well-validated scales to assess interdependence.

### Study 3: Hunting performance

In Study 3, participants played a computerized hunting game under the guise that it had been demonstrated to be strongly predictive of future life success. We altered the parameters of the game such that participants in the failure condition faced a nearly impossible task, while participants in the success condition faced an easily attainable mission. Following this manipulation, participants completed a questionnaire tapping their relative value placed on interdependence versus independence.

#### Participants

We recruited 151 American students (46 male, 105 female) for a laboratory study in exchange for course credit. The sample size was determined by intentionally recruiting more participants than suggested at the time, which would have been 20 participants per cell [39]. Participants were between 18–33 years old (M = 18.79, SD = 1.79). Participants for Study 3 read and signed a consent form printed on paper. This study and informed consent procedure were approved by University of Pittsburgh’s Internal Review Board (PRO12110520).

#### Materials and procedure

Participants were randomly assigned to a success versus failure condition. They were presented with a cover story designed to increase their motivation to perform well on the tasks and the importance of experiencing success/failure on these activities. According to the cover story, “The purpose of this experiment is to explore the evolutionary hunting hypothesis, which states that human evolution was primarily influenced by the activity of hunting. According to this hypothesis, people who make good hunters are the same people who are viewed as intelligent and socially skilled. They are also the people who are successful, make a lot of money, and are happy in their lives. Researchers at MIT have shown that reaching a score of 100 points in 5 minutes on a simple computer hunting game strongly predicts success in these domains, but scores lower than 100 strongly predict failure.”

Participants played a computerized version of the video game Duck Hunt in which participants attempt to shoot flying ducks. Reinforcing the failure or success manipulations, failed trials were followed by an icon of a dog emerging empty handed whereas success trials are followed by the same dog emerging with duck(s) in hand and an expression of happiness. Unbeknownst to participants, we altered the speed of the ducks’ movement such that participants in the failure condition faced a nearly impossible task, while participants in the success condition faced an easily attainable one. Following the study, all participants were debriefed, and no participants reported suspicion regarding this task.

To control for potential impact of the manipulation on mood, participants completed the PANAS measure of positive and negative affect [40] (α for positive affect = .83; α for negative affect = .94).

Participants next completed Singelis’ self-construal scale [41]. This scale has 12 items that measure value placed on interdependence (e.g., My happiness depends on the happiness of those around me, α = .72) and 12 items that measure value placed on independence (e.g., I act the same way no matter who I am with, α = .73). Participants rated their agreement with each item on a 7-point scale from (1) strongly disagree to (7) strongly agree. Because our hypothesis concerns the relative predominance of interdependence versus independence, we subtract the independent score from the interdependent score to determine relative interdependence. The present computation of a continuous measure of the predominance of one motivational orientation over another follows previous research that utilized the same methodology [26–30, 34, 31, 33].

We also included the Meaning in Life Questionnaire [42] as an exploratory pilot test for a different project. This measure did not vary as a function of the manipulation, and will not be discussed further. No other variables were assessed.

#### Results

**Fig 1:**
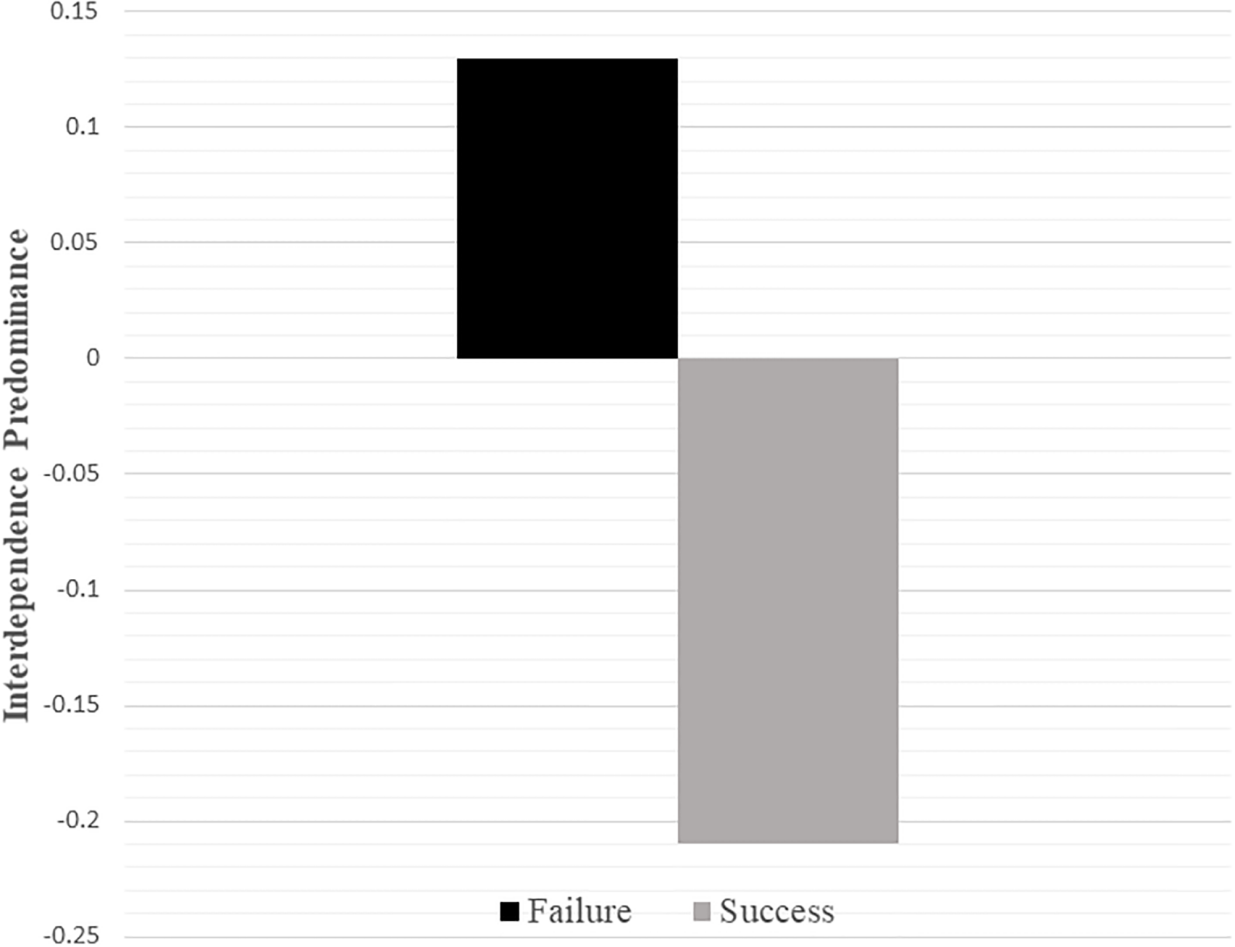
The effect of failure versus success on interdependence predominance (Study 3).

As shown in Fig 1, participants in the failure condition (M = .13, SD = .76) reported higher interdependence predominance than participants in the success condition (M = -.21, SD = .90, t(149) = -2.49, p = .014, d = .41, 95% CI -.61, -.07). Controlling for positive and negative affect produced nearly identical results (p = .015).

Examining independence and interdependence separately, we observed that participants in the failure condition reported lower independence (M = 4.73, SD = .58) than participants in the success condition (M = 5.00, SD = .63, t(149) = 2.79, p = .006, d = .45, 95% CI .08, .47), and that interdependence did not reliably differ across conditions (M_success_ = 4.80, SD_success_ = .61; M_failure_ = 4.86, SD_failure_ = .62; t(149) = -.62, p = .535, d = .10, 95% CI -.26, .14). Controlling for positive and negative affect produced nearly identical results (p = .009; p = .478).

### Study 4: Mathematical and verbal performance

Study 3 provides the first causal evidence for a link between experiences of personal failure and value placed on interdependence. Whereas Study 3 employed a vivid and engaging performance task, our second experiment provided participants with failure or success feedback in the domain of scholastic achievement. Participants then completed a measure of the relative value placed on interdependence and on independence. We used an updated version of the Singelis scale that has the advantage of including additional subscales [43]. While the interdependence/independence distinction reflects a relative focus on the self as part of a collective versus autonomous, the horizontal/vertical distinction reflects a relative concern with inequality and equality. Specifically, the measure includes subscales assessing vertical interdependence, horizontal interdependence, vertical independence, and horizontal independence. Vertical interdependence reflects a perception of the self as embedded in a collective group, but members of the group are represented along a status hierarchy. Horizontal interdependence reflects a perception of the self as embedded in a collective group, and members of the group have similar status. Vertical independence reflects a perception of the self as an autonomous individual in a group in which members have different levels of status. Horizontal independence reflects a perception of the self as an autonomous individual within a group in which members have equal status. Although our primary hypotheses reflected a focus on interdependence predominance, these distinctions allowed us to explore the effects of failure versus success on each of these subscales.

#### Participants

One hundred seventy-four (78 male, 95 female, 1 did not specify) American participants were sampled through Mechanical Turk in exchange for $.40. Participants came from 43 states and were between 19–72 years old (M = 34.20, SD = 11.97). Participants read a consent script on the internet and clicked to continue the survey, indicating that they consented to participate. This study and consent procedure were approved by University of Pittsburgh’s Institutional Review Board (PRO13090365).

#### Materials and procedure

Participants were randomly assigned to a success or failure condition. They were told that the test they would perform assessed their verbal comprehension and mathematical reasoning skills relative to previous participants. Participants then responded to a total of ten questions, five of which were allegedly indicative of their verbal comprehension skills and five concerning their mathematical reasoning skills. All questions were adapted from freely accessible practice Graduate Record Examinations. Next, participants answered three bogus questions in order to distract them from their perceived performance and to lend credibility to the feedback they would receive. Participants specifically indicated whether they had previously held any leadership positions, what their age was, and what their favorite color was. After they clicked the “submit” button, participants were told that the server was calculating their scores and that this would take a couple of seconds. The results were presented after 15 seconds.

Participants who had been randomly assigned to the success condition read: “Your score indicates that you scored higher than 91% of our previously surveyed individuals on the Verbal Comprehension Questionnaire and the Mathematical Reasoning Questionnaire. This means that you possess more skills relevant to understanding and analyzing written materials as well as that your ability to apply quantitative skills and solve problems effectively is more developed than in 91% of other individuals.” Participants in the failure condition read: “Your score indicates that you scored lower than 81% of our previously surveyed individuals on the Verbal Comprehension Questionnaire and the Mathematical Reasoning Questionnaire. This means that you possess less skills relevant to understanding and analyzing written materials as well as that your ability to apply quantitative skills and solve problems effectively is less developed than in 81% of other individuals.”

Participants completed the PANAS affect measure [40] (α for positive affect = .90; α for negative affect = .90). As a measure of their relative independent versus interdependent orientation, participants completed a frequently used measure of collectivistic and individualistic orientations [43]. This measure was developed by the scale authors from the set of items in the measure we used in Study 3. We used this scale because the items map particularly well onto the relative value placed on interdependence and independence which is of central interest in the present research, and because this scale is a more recent update to the scale used in Study 3. Participants indicated their agreement with eight items tapping interdependence (e.g., “I feel good when I cooperate with others”) and eight items tapping independence (e.g., “I rely on myself most of the time”) on a 7-point scale from Strongly Disagree (1) to Strongly Agree (7). We aggregated across the interdependence items (α = .79) and independence items (α = .72) and created an index of interdependent predominance over independence.

We also included the Meaning in Life Questionnaire [42] and the [44] Self-Esteem Questionnaire as exploratory pilot tests. Search for meaning was significantly higher in the failure than the success condition (t = 2.15, p = .033). Presence of meaning and self-esteem did not vary as a function of condition. These measures will not be discussed further. No other variables were assessed.

#### Results

**Fig 2:**
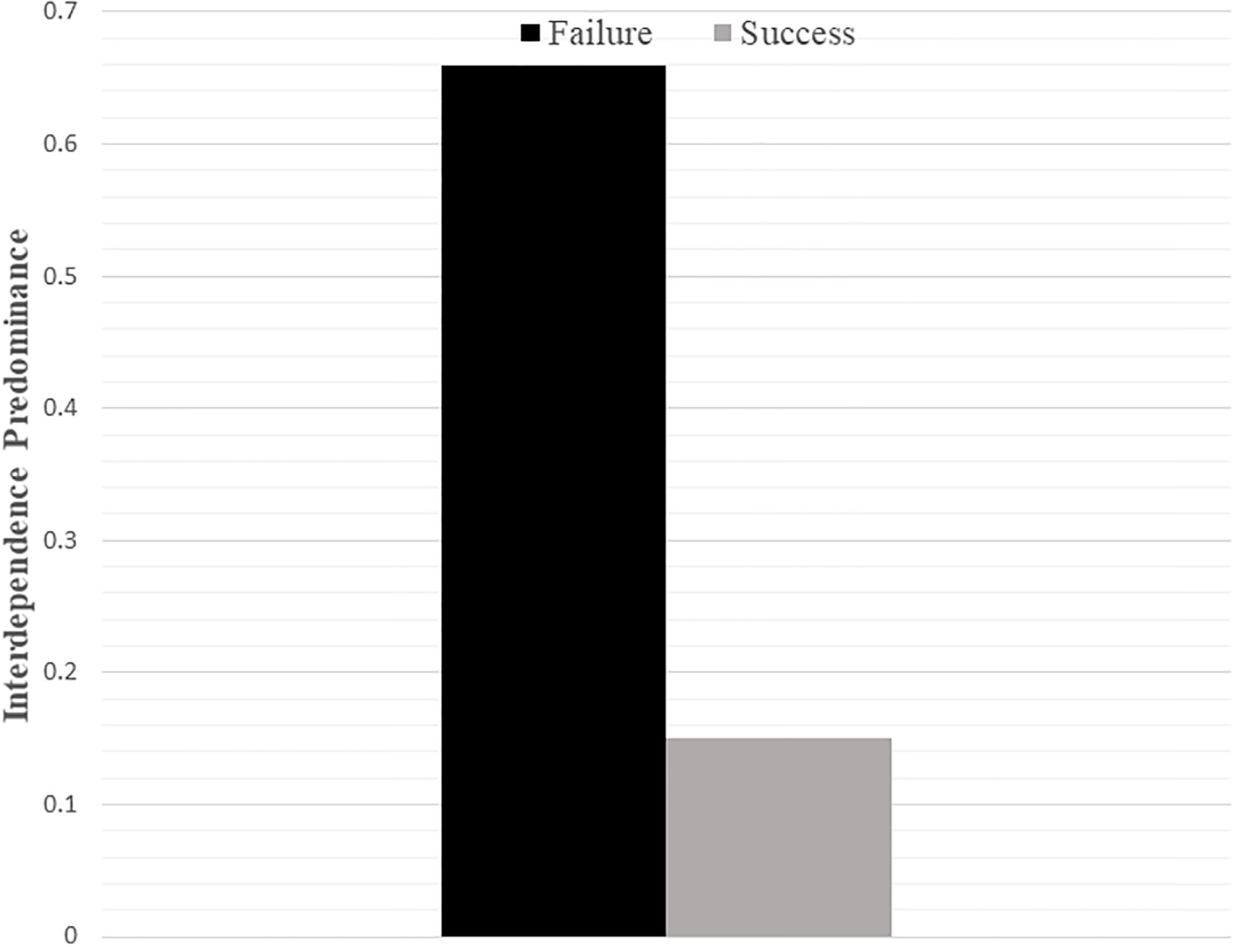
The effect of failure versus success on interdependence predominance (Study 4).

We tested whether interdependence predominance differed across the failure versus success conditions. As shown in Fig 2, interdependence predominance was higher in the failure (M = .66, SD = 1.21) as compared to success condition (M = .15, SD = 1.29, t(172) = 2.70, p = .008, d = .41, 95% CI .13, .88). Controlling for affect did not alter the findings (p = .003).

Examining independence and interdependence separately, we observed that participants in the failure condition showed a trend toward lower independence (M = 4.50, SD = .95) than participants in the success condition (M = 4.72, SD = .82, t(172) = -1.62, p = .108, d = .25, 95% CI -.49, .05), and that participants in the failure condition reported significantly higher interdependence (M = 5.16, SD = .91) than in the success condition (M = 4.87, SD = .91; t(172) = 2.10, p = .037, d = .32, 95% CI .02, .56). Controlling for positive and negative affect did not influence the reliability of the results (p = .409 for independence; p = .001 for interdependence).

Examining each of the four subscales separately, we observed that participants in the failure condition reported significantly lower horizontal individualism (M = 4.98, SD = 1.21) than participants in the success condition (M = 5.49, SD = .94, t(172) = -3.12, p = .002, d = .47, 95% CI -.84, -.19). Participants in the failure condition reported significantly higher horizontal collectivism (M = 5.18, SD = .92) than participants in the success condition (M = 4.89, SD = .97, t(172) = 2.01, p = .046, d = .31, 95% CI .01, .57). Vertical individualism did not differ between the failure condition (M = 4.03, SD = 1.37) and success condition (M = 3.95, SD = 1.17, t(172) = .41, p = .685, d = .06, 95% CI -.30, .46). Vertical collectivism did not differ between the failure condition (M = 5.15, SD = 1.25) and the success condition (M = 4.85, SD = 1.22, t(172) = 1.56, p = .120, d = .21, 95% CI -.08, .66). Controlling for positive and negative affect led to a marginal effect for horizontal individualism (p = .06), and did not alter the reliability of horizontal collectivism (p = .001) or vertical individualism (p = .661). When controlling for positive and negative affect, vertical collectivism was significantly higher in the failure condition (M = 4.15, SD = 1.25) than in the success condition (M = 4.85, SD = 1.22), F(1, 170) = 5.31, p = .022.

#### Discussion

Results of Studies 3 and 4 provide causal evidence that failure (versus success) increases interdependence predominance. These findings were observed across different tasks (a game and a scholastic test), providing conceptual replication of the findings, and attesting to their generalizability and robustness.

### General discussion

Human nature pits the relative value placed on interdependence with others against the value of individual independence. From birth through adulthood, people are faced with the need to balance individual needs with social concerns. The present research investigated the conditions that foster a relative orientation toward greater interdependence versus independence. We found that failure causes a shift in perspective such that the person orients his or her thinking to focus on ways s/he is interdependent with others. Thus, it seems that when people experience personal hardship, they may be more likely to turn to their close others, community, tribe, nation, and religion to help them feel stronger and more valuable. In contrast, when they experience personal success and prosperity, they may be more likely to claim personal responsibility and exhibit independence. These findings have implications for understanding the experience of personal failure and its psychological consequences, and call for more research examining the psychological consequences of individual and societal-level periods of hardship and prosperity.

Now that experimental evidence confirms the causal role of failure (success) in contributing to interdependence (independence), we may be able to better understand why some individuals and cultures value independence more than others. We interpret the present findings as suggesting that the experience of personal success, wealth, prosperity, and esteem is likely causing individuals to value independence. In their theory of cultural development, Inglehart and Oyserman [13] suggest that increases in economic development drive cultures to value self-expression and personal choice. This form of success leads cultures to shift toward greater value placed on independence. Indeed, as nations have become more prosperous, the value placed on independence has also increased [12, 45, 13–14, 10]. During times of hardship, however, we would expect cultures to shift toward a more interdependent orientation. Historical examples seem to support this. For example, the Great Depression shifted values toward a greater focus on interdependence, propelling acceptance of the New Deal in the United States. We hope that future research will undertake a detailed analysis of historical trends and their resulting psychological consequences, and think this would be an important contribution.

Social coordination through interdependence can have pro- or anti-social outcomes. Interdependence increases a person’s willingness to sacrifice for the sake of the group [6, 46], and groups tend to reward such sacrifices [47]. Given the appropriate ideology, an interdependent orientation fosters prosocial behavior in which people band together to rise to challenges of economic downturns, natural disasters, or the spread of disease. Yet the interdependent impulse might be taken in a destructive direction as well and promote anti-social behavior against outgroups, the revocation of human rights, and/or the fanning of inter-group violence [48, 4, 49]. Failure may thus augment interdependent bonds among individuals and ready them to embark on social action whose specific impact can be ideologically driven for good and for ill. Understanding this process, enlightened by the present findings, may significantly inform our efforts to ensure that the former rather than the latter be the case [50–51].

Each of the present studies investigated success or failure on a personal goal. An important question concerns the generalizability of this effect across types of success and failure, and the boundary conditions of this effect. While the present studies are not able to address this point, some additional research from our lab may hint at potential boundary conditions. We conducted four studies that manipulated whether participants were asked to recall a previous failure (vs. success or control). In one of these studies, we found a marginal effect (*p* = .054) in the expected direction, but reliable differences did not emerge in the other three studies. These findings suggest to us that the experience of personal failure should be relatively vivid and felt in the moment in order to reliably produce shifts in interdependence predominance. Future research could continue to explore the boundary conditions of these effects.

While the present research focused on failure on a personal goal, other research has focused on failure or suffering among groups [52–54]. Future research could compare success versus failure on personal and group goals. Future theoretical work could integrate across these different approaches.

While the present research investigated the relative predominance of the interdependence motive as compared to the independence motive, readers may wonder whether these two motives are necessarily antagonistic. Although motives may come into conflict with one another, resulting in the momentary privileging of one over the other, a useful strategy of resolving goal conflict may be to find a method of pursuing both motives at the same time, possibly via *multifinal* means [55–57, 34]. Indeed, at least eight strategies for concurrently pursuing independence and interdependence motives have been identified in the literature [58]. Thus, future research should investigate the consequences of success and failure for the specific means adopted to support pursuit of interdependence and independence motives.

An important limitation of the first two studies in this package was the single-item measure of interdependence. This limitation is offset by the of well-validated, multi-item, scales in the subsequent two studies. The conclusions reached here remain tentative until they are replicated and extended via additional research. Of particular interest would be demonstrating the replicability and generalizability of success versus failure manipulations on measures of interdependence predominance, and extensions of this work to personal and historical covariance between prosperity/hardship and markers of interdependence predominance.

## References

[1] Hofstede G. Culture’s consequences. 1980 Beverly Hills Sage.

[2] Kruglanski AW, Bélanger JJ, Gelfand M, Gunaratna R, Hettiarachchi M, Reinares F, et al Terrorism—A (self) love story: Redirecting the significance quest can end violence. American Psychologist. 2013 10;68(7):559–575. 10.1037/a0032615 24128318

[3] Kruglanski AW, Chen X, Dechesne M, Fishman S, Orehek E. Fully committed: Suicide bombers' motivation and the quest for personal significance. Political Psychology. 2009 6 1;30(3):331–57.

[4] Kruglanski AW, Orehek E. The role of the quest for personal significance in motivating terrorism In Social Conflict and Aggression (pp. 153–164). New York: Psychology Press 2011

[5] Kruglanski AW, Jasko K, Chernikova M, Dugas M, Webber D. To the fringe and back: Violent extremism and the psychology of deviance. American Psychologist. 2017 4;72(3):217–230. 10.1037/amp0000091 28383975

[6] Orehek E, Sasota JA, Kruglanski AW, Dechesne M, Ridgeway L. Interdependent self-construals mitigate the fear of death and augment the willingness to become a martyr. Journal of Personality and Social Psychology. 2014 8;107(2):265–275. 10.1037/a0036675 24819868

[7] Ainsworth MD, Bell SM. Attachment, exploration, and separation: Illustrated by the behavior of one-year-olds in a strange situation. Child development. 1970 3 1:49–67. 5490680

[8] Schachter S. The psychology of affiliation: Experimental studies of the sources of gregariousness. Stanford University Press; 1959.

[9] Diener E, Diener M. Cross-cultural correlates of life satisfaction and self-esteem In Culture and well-being 2009 (pp. 71–91). Springer Netherlands.

[10] Santos HC, Varnum ME, Grossmann I. Global increases in individualism. Psychological science. 2017 9;28(9):1228–39. 10.1177/0956797617700622 28703638

[11] Hofstede G. Cultures and organizations: Software of the mind 1991 London/New York McGraw-Hill.

[12] Inglehart R. Modernization and postmodernization: Cultural, economic, and political change in 43 societies. Princeton University Press; 1997.

[13] Inglehart R, Oyserman D. Individualism, autonomy and self-expression: The human development syndrome. International Studies in Sociology and Social Anthropology 2004 In Comparing Cultures, Dimensions of Culture in a Comparative Perspective. Leiden, The Netherlands Brill.

[14] Welzel C, Inglehart R, Kligemann HD. The theory of human development: A cross‐cultural analysis. European Journal of Political Research. 2003 5 1;42(3):341–79.

[15] Arrindell WA, Hatzichristou C, Wensink J, Rosenberg E, van Twillert B, Stedema J, et al Dimensions of national culture as predictors of cross-national differences in subjective well-being. Personality and Individual differences. 1997 7 1;23(1):37–53.

[16] Diener E, Diener M, Diener C. Factors predicting the subjective well-being of nations. Journal of Personality and Social Psychology. 1995 11;69(5):851–864. 7473035 10.1037//0022-3514.69.5.851

[17] Fishbach A, Ferguson MJ. The goal construct in social psychology In Kruglanski AW, Higgins ET (Eds.), Social Psychology: handbook of Basic Principles (pp. 490–515). New York: Guildford Press 2007.

[18] Gollwitzer PM, Heckhausen H, Steller B. Deliberative and implemental mind-sets: Cognitive tuning toward congruous thoughts and information. Journal of Personality and Social Psychology. 1990 12;59(6):1119–1127.

[19] Kruglanski AW, Shah JY, Fishbach A, Friedman R, Chun WY, Sleeth-Keppler D. A theory of goal systems. Advances in Experimental Social Psychology. 2002 12 31;34:331–78.

[20] Lewin K. Contributions to psychological theory The conceptual representation and the measurement of psychological forces. Durham, NC, US: Duke University Press 1938.

[21] Orehek E, Vazeou-Nieuwenhuis A. Sequential and concurrent strategies of multiple goal pursuit. Review of General Psychology. 2013 9;17(3):339–349.

[22] Carver CS, Scheier MF. On the self-regulation of behavior. Cambridge University Press; 2001.

[23] Förster J, Liberman N, Friedman RS. Seven principles of goal activation: A systematic approach to distinguishing goal priming from priming of non-goal constructs. Personality and Social Psychology Review. 2007 8;11(3):211–33. 10.1177/1088868307303029 18453462

[24] Gollwitzer PM, Schaal B. Metacognition in action: The importance of implementation intentions. Personality and social psychology review. 1998 5;2(2):124–36. 10.1207/s15327957pspr0202_5 15647140

[25] Shah JY, Friedman R, Kruglanski AW. Forgetting all else: on the antecedents and consequences of goal shielding. Journal of Personality and Social Psychology. 2002 12;83(6):1261 12500810

[26] Bohns VK, Lucas GM, Molden DC, Finkel EJ, Coolsen MK, Kumashiro M, et al Opposites fit: Regulatory focus complementarity and relationship well-being. Social Cognition. 2013 2;31(1):1–4.

[27] Cesario J, Grant H, Higgins ET. Regulatory fit and persuasion: Transfer from" feeling right". Journal of Personality and Social Psychology. 2004 3;86(3):388–404. 10.1037/0022-3514.86.3.388 15008644

[28] Higgins ET. Culture and personality: Variability across universal motives as the missing link. Social and Personality Psychology Compass. 2008 3 1;2(2):608–34.

[29] Higgins ET, Pierro A, Kruglanski AW. Re-thinking Culture and Personality-Chapter 8: How Self-Regulatory Universals Create Cross-Cultural Differences In Sorrentino R.M. (Ed.), Handbook of motivation and cognition within and across cultures 2008 (pp. 102–143). New York Guilford Press.

[30] Hong J, Lee AY. Be fit and be strong: Mastering self-regulation through regulatory fit. Journal of Consumer Research. 2007 8 20;34(5):682–95.

[31] Scholer AA, Ozaki Y, Higgins ET. Inflating and deflating the self: Sustaining motivational concerns through self-evaluation. Journal of Experimental Social Psychology. 2014 3 1;51:60–73.

[32] Avnet T, Higgins ET. Locomotion, assessment, and regulatory fit: Value transfer from “how” to “what”. Journal of Experimental Social Psychology. 2003 9 1;39(5):525–30.

[33] Zee KS, Cavallo JV, Flores AJ, Bolger N, Higgins ET. Motivation moderates the effects of social support visibility. Journal of Personality and Social Psychology. 2018 5;114(5):735–765. 10.1037/pspi0000119 29376663

[34] Orehek E, Mauro R, Kruglanski AW, van der Bles AM. Prioritizing association strength versus value: the influence of self-regulatory modes on means evaluation in single goal and multigoal contexts. Journal of Personality and Social Psychology. 2012 1;102(1):22–31. 22514798 10.1037/a0025881

[35] Gardner WL, Gabriel S, Lee AY. “I” value freedom, but “we” value relationships: Self-construal priming mirrors cultural differences in judgment. Psychological Science. 1999 7;10(4):321–6.

[36] Oyserman D, Lee SW. Does culture influence what and how we think? Effects of priming individualism and collectivism. Psychological Bulletin. 2011 134, 311–342. 10.1037/0033-2909.134.2.311 18298274

[37] Trafimow D, Triandis HC, Goto SG. Some tests of the distinction between the private self and the collective self. Journal of Personality and Social Psychology. 1991 5;60(5):649–655.

[38] Efron B, Tibshirani RJ. Permutation tests In An introduction to the bootstrap 1993 1 1 (pp. 202–219). Springer US.

[39] Simmons JP, Nelson LD, Simonsohn U. False-positive psychology: Undisclosed flexibility in data collection and analysis allows presenting anything as significant. Psychological Science. 2011 11;22(11):1359–66. 10.1177/0956797611417632 22006061

[40] Watson D, Clark LA, Tellegen A. Development and validation of brief measures of positive and negative affect: the PANAS scales. Journal of Personality and Social Psychology. 1988 6;54(6):1063 3397865 10.1037//0022-3514.54.6.1063

[41] Singelis TM. The measurement of independent and interdependent self-construals. Personality and Social Psychology Bulletin. 1994 10;20(5):580–91.

[42] Steger MF, Frazier P, Oishi S, Kaler M. The meaning in life questionnaire: Assessing the presence of and search for meaning in life. Journal of Counseling Psychology. 2006 1;53(1):80–93.

[43] Singelis TM, Triandis HC, Bhawuk DP, Gelfand MJ. Horizontal and vertical dimensions of individualism and collectivism: A theoretical and measurement refinement. Cross-Cultural Research. 1995 8;29(3):240–75.

[44] Rosenberg M. Society and the adolescent self-image. Princeton, NJ: Princeton University Press; 1965.

[45] Inglehart R, Baker WE. Modernization, cultural change, and the persistence of traditional values. American Sociological Review. 2000 2 1:19–51.

[46] Swann WB Jr, Buhrmester MD, Gómez A, Jetten J, Bastian B, Vázquez A, et al What makes a group worth dying for? Identity fusion fosters perception of familial ties, promoting self-sacrifice. Journal of personality and social psychology. 2014 6;106(6):912 10.1037/a0036089 24841096

[47] Willer R. Groups reward individual sacrifice: The status solution to the collective action problem. American Sociological Review. 2009 2;74(1):23–43.

[48] Orehek E, Fishman S, Dechesne M, Doosje B, Kruglanski AW, Cole AP, et al Need for closure and the social response to terrorism. Basic and applied social psychology. 2010 11 18;32(4):279–90.

[49] Kruglanski AW, Orehek E. The need for certainty as a psychological nexus for individuals and society. Extremism and the psychology of uncertainty. 2012:3–18.

[50] Kruglanski AW, Gelfand MJ, Bélanger JJ, Sheveland A, Hetiarachchi M, Gunaratna R. The psychology of radicalization and deradicalization: How significance quest impacts violent extremism. Political Psychology. 2014 2 1;35(S1):69–93.

[51] Orehek E, Vazeou-Nieuwenhuis A. Understanding the terrorist threat: Policy implications of a motivational account of terrorism. Policy Insights from the Behavioral and Brain Sciences. 2014 10;1(1):248–55.

[52] Bastian B, Jetten J, Ferris LJ. Pain as social glue: Shared pain increases cooperation. Psychological science. 2014 11;25(11):2079–85. 10.1177/0956797614545886 25193943

[53] De Cremer D, Van Dijk E. Reactions to group success and failure as a function of identification level: A test of the goal-transformation hypothesis in social dilemmas. Journal of Experimental Social Psychology. 2002 9 1;38(5):435–42.

[54] Von Dawans B, Kirschbaum C, Heinrichs M. The Trier Social Stress Test for Groups (TSST-G): A new research tool for controlled simultaneous social stress exposure in a group format. Psychoneuroendocrinology. 2011 5 1;36(4):514–22. 10.1016/j.psyneuen.2010.08.004 20843608

[55] Chun WY, Kruglanski AW, Sleeth-Keppler D, Friedman RS. Multifinality in implicit choice. Journal of Personality and Social Psychology. 2011 11;101(5):1124–1137. 10.1037/a0023778 21574723

[56] Köpetz C, Faber T, Fishbach A, Kruglanski AW. The multifinality constraints effect: how goal multiplicity narrows the means set to a focal end. Journal of Personality and Social Psychology. 2011 5;100(5):810–826. 10.1037/a0022980 21381854

[57] Kruglanski AW, Köpetz C, Bélanger JJ, Chun WY, Orehek E, Fishbach A. Features of multifinality. Personality and Social Psychology Review. 2013 2;17(1):22–39. 10.1177/1088868312453087 22854862

[58] Hornsey MJ, Jetten J. The individual within the group: Balancing the need to belong with the need to be different. Personality and Social Psychology Review. 2004 8;8(3):248–64. 10.1207/s15327957pspr0803_2 15454348

